# A clinical approach to the assessment and management of co-morbid eating disorders and substance use disorders

**DOI:** 10.1186/1471-244X-13-289

**Published:** 2013-11-07

**Authors:** Claire Gregorowski, Soraya Seedat, Gerhard P Jordaan

**Affiliations:** 1 Department of Psychiatry, Faculty of Health Sciences, Stellenbosch University, Cape Town, South Africa

## Abstract

**Introduction:**

Research has shown that eating disorder (ED) patients who abuse substances demonstrate worse ED symptomatology and poorer outcomes than those with EDs alone, including increased general medical complications and psychopathology, longer recovery times, poorer functional outcomes and higher relapse rates. This article provides a broad overview of the prevalence, aetiology, assessment and management of co-morbid EDs and substance use disorders (SUDs).

**Review:**

The co-occurrence of EDs and SUDs is high. The functional relationship between EDs and SUDs vary within and across ED subtypes, depends on the class of substance, and needs to be carefully assessed for each patient. Substances such as caffeine, tobacco, insulin, thyroid medications, stimulants or over the counter medications (laxatives, diuretics) may be used to aid weight loss and/or provide energy, and alcohol or psychoactive substances could be used for emotional regulation or as part of a pattern of impulsive behaviour. A key message conveyed in the current literature is the importance of screening and assessment for co-morbid SUDs and EDs in patients presenting with either disorder. There is a paucity of treatment studies on the management of co-occurring EDs and SUDs. Overall, the literature indicates that the ED and SUD should be addressed simultaneously using a multi-disciplinary approach. The need for medical stabilization, hospitalization or inpatient treatment needs to be assessed based on general medical and psychiatric considerations. Common features across therapeutic interventions include psycho-education about the aetiological commonalities, risks and sequelae of concurrent ED behaviours and substance abuse, dietary education and planning, cognitive challenging of eating disordered attitudes and beliefs, building of skills and coping mechanisms, addressing obstacles to improvement and the prevention of relapse. Emphasis should be placed on building a collaborative therapeutic relationship and avoiding power struggles. Cognitive behavioural therapy has been frequently used in the treatment of co-morbid EDs and SUDs, however there are no randomized controlled trials. More recently evidence has been found for the efficacy of dialectical behavioural therapy in reducing both ED and substance use behaviours.

**Conclusion:**

Future research would benefit from a meta-analysis of the current research in order to better understand the relationships between these two commonly co-occurring disorders.

## Introduction

The understanding and management of eating disorders (EDs) presents challenges to researchers and clinicians alike. Complicating factors include patients’ reluctance to seek treatment because of psychological investment in maintaining the disorder, high rates of co-morbidity with other Axis I and II disorders and variations in symptom presentation and aetiological factors both within and across ED subtypes [1-3]. Co-morbid substance use disorder (SUD) is commonly found with EDs and can lead to increased general medical complications and psychopathology, longer recovery times, poorer functional outcomes and higher relapse rates [4-7]. This article considers the prevalence of co-morbid EDs and SUDs, the types of substances used and their role and function in ED pathology before outlining assessment and diagnostic considerations and management strategies.

## Review

### Epidemiology

#### Prevalence

The rates of co-morbid eating EDs and SUDs reported in the literature are high. These rates do vary across studies however, in part due to the heterogeneity of definitions of SUDs that have been applied. For example, only some studies distinguish between substance use, abuse and dependence or disorder. Studies have reported that up to 50% of patients with an eating disorder will abuse alcohol or an illicit substance, compared with 9% of the general population, and that 35% of alcohol or illicit substance abusers have an eating disorder where the general population prevalence of an eating disorder is 3% [8]. In a more recent review of the literature, rates of between 17% and 46% were reported [7]. In adolescent populations exhibiting eating disordered behaviour, rates of substance use and abuse has also been found to be 20-40% greater than that of normal weight peers [9].

Traditionally, Bulimia Nervosa (BN) has had the strongest reported association with substance use compared with Anorexia Nervosa (AN) [6,10-14]. In a meta-analysis of the literature, Calero-Elvira et al. (2009) found a “significant but negligible” increase in substance use amongst those with an ED compared with healthy controls. They reported the highest prevalence of substance use in those with BN purging type followed by those with Binge Eating Disorder (BED), compared with the healthy population, whereas those with AN restricting type had lower levels of drug use than healthy controls [15].

However, some recent studies have documented that co-morbid AN and SUDs may be more prevalent than previously thought, particularly for those with AN with bulimic features [10,13,14,16]. For example, Root et al. (2010b) found that women with AN, BN and a lifetime diagnosis of AN *and* BN (ANBN) all had increased risk of substance use compared with controls, with ED subgroups with bulimic features being at increased risk for alcohol use/dependence compared with the restricting AN group (R-AN), and the R-AN group having similar levels of illicit drug use as the binge/purge AN group [14]. Baker et al. (2010) found no significant difference in prevalence rates of SUD between AN patients and patients with BN, however this study did not differentiate between AN subtypes [16]. Root et al. (2010a) found that substance abuse/dependence was most common among those with a lifetime diagnosis of AN *and* BN (ANBN), least common among those with restricting AN, and the presence of purging behaviour was more highly associated with SUDs than non-purging behaviour [13]. Thus, substance use by patients with AN is more common than previously thought, ED patients with bulimic features appear to be at highest risk for alcohol use disorders, and evidence for prevalence of drug use amongst R-AN patients is mixed. Some studies have documented specific associations between eating disorder sub-types and varying patterns of substance use, but data is inconsistent.

#### Substances of abuse

##### Alcohol

Alcohol abuse/dependence has been found to be more common in those with BN, binge eating (BE), or those with AN who engage in binging and/or purging behaviour [17,18]. Dansky et al. (2000) reported higher levels of alcohol dependence in women with BN compared to those without, but found higher levels of alcohol *abuse* only when the presence of depression and PTSD was controlled for, indicating that these conditions may influence or mediate the relationship between BN and alcohol abuse. They concluded that 31% of women with BN also have a history of alcohol abuse and 13% have a history of alcohol dependence [19]. However studies do differ. For example, Franko et al. (2005) reported prevalence rates of 27% of lifetime alcohol use disorder (AUD) in their sample of ED patients. Levels of alcohol abuse/dependence did not differ significantly in those with AN compared with BN. However, of the AN group reporting an AUD 72.7% were AN binge purging type, which may explain this difference. Krug et al. (2008) found no significant difference in lifetime and current alcohol use between ED patients and a healthy control group [11,20]. Within the ED group, those with a diagnosis of Eating Disorders Not Otherwise Specified (EDNOS) had higher levels of current and lifetime alcohol use compared with other ED types, including BN [11]. This can be explained by the fact that many studies do not include patients diagnosed with EDNOS. This underscores the need for research on this commonly diagnosed ED subtype [11]. Overall, the data indicate that alcohol abuse/dependence is more commonly found in ED subgroups with bulimic features.

Where differences have been found, binge drinking (associated with alcohol abuse) seems to be more highly associated with BN than frequency of use (associated with alcohol dependence). In a recent study, Piran and Robinson (2011) found in a non-clinical sample that the cluster of binging, dieting and purging behaviour was most strongly associated with binge drinking (and cocaine use) but not with the frequency of alcohol use [21]. Stewart et al. (2000) also found that dietary restraint was associated with the quantity of alcohol consumption (binge drinking) in women with BN, but not with the frequency [22].

Several hypotheses have been suggested to explain this differential relationship. Alcohol abuse and the ED may be part of more general behaviour dysregulation including impulsivity. Alcohol could be used as a form of emotional regulation in ED women. Alternatively, increased binge drinking may be the result of attempts to restrict alcohol intake (because of the high calorie levels in alcohol) followed by alcohol binges [18,22]. Studies have shown that restriction of a substance such as food or alcohol leads to increased reinforcement value for that or other substances [23].

Alcohol use may also be a risk factor for the development of an eating disorder [24,25]. Some studies have indicated an increased prevalence of ED behaviours in adolescents using alcohol, with frequency and severity of alcohol consumption found to be positively associated with number of ED symptoms [24]. Similarly, Piran and Gadalla (2006), in a nationally representational study of Canadian women, reported an association between risk for developing an eating disorder and lifetime alcohol dependence [26]. In contrast, Franko et al. (2005) found that having an ED was more likely to lead to the development of an AUD than the reverse. They report depression and over concern with body image and vomiting as the main risk factors for developing a co-morbid AUD in women with AN [20]. The latter study includes patients diagnosed with an ED, rather than risk for ED or subclinical ED symptoms. This may explain the greater impact of EDs on SUDs reported by Franko et al. (2005), in contrast to the inverse relationship suggested by the previous two studies. Further investigation of the pathobiological and temporal relationship between these two disorders is needed.

##### Caffeine and tobacco

Caffeine is used as an appetite suppressant or to provide energy and can be consumed through coffee or diet drinks [8]. Similarly, tobacco is used as a weight loss aid, not only because it suppresses appetite, but because it can help to distract from thoughts about food [8]. Distorted body image is associated with increased risk of a caffeine abuse disorder and tobacco use has been associated with greater levels of eating disorder pathology [8,13,16].

Baker et al. (2010) reported in their study that caffeine and tobacco were the most frequently used substances, and women with AN were more likely to have a caffeine disorder and to smoke cigarettes (26% and 52% respectively), compared with those with BN (23% and 45%). However, this difference was found to be non-significant and AN in this study was broadly defined. [16]. Krug et al. (2008) reported that women with an ED (particularly those with BN or AN-binging/purging type) were more likely to smoke, and smoked more frequently as a way of controlling weight, than healthy controls [11]. In contrast, Root et al. (2010b) reported no difference in levels of tobacco use across AN subtypes, including an AN and BN lifetime group [14]. Thus, tobacco and caffeine use is greater amongst ED patients compared with controls but reported prevalence rates across ED subgroups are somewhat inconsistent.

##### Illicit substances (amphetamines/stimulants/cannabis/opiates)

Amphetamines may be used as appetite suppressants in order to aid weight loss [15,16]. Psychoactive substances may also be used to help regulate painful affect [27]. In a review of the literature, Holderness et al. (2004) found that rates of amphetamine use were found to be higher in patients with AN compared with BN [17]. Piran and Robinson (2006, 2011) found associations between dieting and purging behaviour (without binging) and amphetamine use [18,21]. An association has previously been shown between dieting and bingeing and amphetamine use, however Calero-Elvira et al. (2009) did not find increased use of these classes of substance in their ED group (combined BN, BED and AN) compared with the general population [15]. Amphetamine use may therefore be associated with dieting and purging behaviour rather than binging behaviour, which might be expected given its role in aiding weight loss, however data is inconsistent. A recent study by Jeffers et al. (2013) reported on the wide use of prescribed stimulants (usually used in the treatment of ADHD) as weight loss aids in a group of 705 undergraduate students [28].

Calero-Elvira et al. (2009) reported increased prevalence of cannabis and opiates in those with an ED (subgroups combined) compared with controls, however AN restricting type did not demonstrate significant combined drug use compared to controls [15]. Root et al. (2010) found increased cannabis use in ED patients compared to a control group and reported comparative prevalence rates across ED subgroups for this class of substance. They further reported that cannabis was the most frequently used drug among AN patients, and hypothesized that this reflected norms of the general population where cannabis was the most frequently used illicit substance [14]. Calero-Elvira et al. (2009) postulated that this class of illicit substances may be also be used to cancel out the restlessness associated with stimulant use [15]. In sum, illicit drug use seems generally more common in ED populations compared to healthy controls; they may be used as weight-loss aids, to regulate affect or to counteract the effects of other substances. More data is needed to clarify differential relationships between the types of illicit drugs and ED subgroups.

##### Over the counter or prescription medications (laxatives/diuretics/diet pills/thyroid medications/insulin)

Laxatives are used as a means of weight loss. They are thought to be the most commonly abused substances by those with an eating disorder, with prevalence rates of up to 75% being reported in this population [12]. According to Mitchell et al. (1997), ED patients who misuse laxatives have higher levels of co-morbid psychopathology than those who do not, leading to treatment complications [12]. Pryor et al. (1996) found that laxative use predicted higher levels of perfectionism and avoidant personality traits [29]. Bryant-Waugh et al. (2006) reported that 26.4% of their sample of 201 outpatients with an eating disorder diagnosis used laxatives as a means of losing weight, and these patients were identified as having more severe clinical presentations (eating disorder symptomatology and other psychopathology) that were independent of the ED type [30].

Stimulant type laxatives are most commonly used and desensitization leads to increased levels of usage in individuals [12]. Laxative misuse has been found to be an ineffective weight-loss mechanism, and the abuse of laxatives results in medical complications such as chronic diarrhea, disturbances in electrolyte and acid–base balance, reflex peripheral edema, constipation, impairment in colon functioning and nephrolithiasis (kidney stones), among others [12,31,32]. An association has been found between laxative use and use of diuretics and diet pills, however diet pills are less frequently used compared with laxatives [12,33]. Diuretics are commonly used as a method of purging in patients with BN, often resulting in severe electrolyte imbalances [34]. The use of multiple forms of purging methods has been associated with increased eating disorder severity, whereas purging frequency has been shown to predict other forms of psychopathology such as depression, impulsivity, anxiety and personality disorders [35]. ED patients may also abuse thyroid replacement hormones in order to increase metabolism and support weight-loss, leading to increased risk for cardiovascular and metabolic problems, diabetes and hypertension [36]. Patients with diabetes may also reduce or manipulate insulin use (particularly after binge eating) in order to facilitate weight loss through calorie loss [36,37] (Additional file 1: Table S1).

#### Aetiological and risk factors

The aetiological factors contributing towards co-morbid eating and substance abuse disorders are best understood from a biopsychosocial perspective. Aetiological theories of co-morbidity include biological (e.g. genetic and familial risk factors), addiction and behavioural models, underlying personality factors (such as chronic dysregulation, increased impulsivity and novelty seeking), other co-morbid psychopathology, as well as environmental factors [7,10].

##### Biological factors

Biological models implicate common disturbances in neurotransmitter function in dopamine, serotonin, gamma aminobutyric acid, and endogenous opiate systems across both EDs and SUDs [7,38]. Evidence for this model includes the similarity in physical symptoms across these disorders and the links that have been shown between food deprivation and the increased “biologically reinforcing effects of substances” [7].

With regards to an inherited genetic predisposition, studies have shown independent genetic heritabilities for SUDs and EDs, however, there is insufficient evidence from either twin or family studies for a shared genetic link [7,14,16,39]. With regards to twin studies, Kendler and colleagues (1995) researched the genetic and environmental risk factors for six psychiatric disorders including BN and alcohol abuse. They concluded that vulnerability for BN and alcohol abuse was influenced by separate genetic factors. While BN may share risk factors with other psychiatric disorders, the majority of genetic factors affecting risk for alcohol abuse are specific to the disorder. In addition, co-morbidity was affected by a combination of genetic and environmental (family and individual) influences [40]. Wade et al. (2004) found, in one twin study, that psychoactive substance use but not abuse or dependence was associated with increased risk for BN in the other and concluded that the frequent co-morbidity between BN and SUDs was not due to shared familial risk factors, but a combination of genetic and environmental influences. They further reported that risk for BN was only associated with risk for neuroticism and novelty seeking in male siblings. Further, novelty-seeking was associated with psychoactive substance use in this subgroup. They suggested that there may be a familial predisposition with respect to BN and novelty seeking in men, which may manifest in psychoactive substance use [41]. Baker et al. (2010) reported significant co-variance between EDs and SUDs which may be attributable to environmental and/or genetic factors, but identified genetic factors as the more important contributor to the overlap [16]. Similarly, Slane et al. (2012) found a significant correlation in genetic factors between bulimic behaviours (particularly compensatory behaviours, followed by binge eating) and co-morbid alcohol use, providing evidence for genetic or heritable links between these two types of disordered behaviour [42].

Considering family studies, Lilenfeld et al. (1998) found no common familial link between BN and substance use disorders [43]. Redgrave et al. (2007) reported higher rates of binging, vomiting, laxative and diet pill use across the lifetime, increased ED psychopathology (i.e. higher scores on several domains of the Eating Disorders Inventory −2), and higher rates of alcohol use amongst ED patients with a first-degree relative with an alcohol abuse disorder compared to those without. The authors concluded that having a first degree relative who abused alcohol was likely to exacerbate (rather than cause) ED psychopathology [39].

##### Addictions model

The addictions model purports that both EDs and SUDs are based on chemical dependency with similar genetic, familial, personality and socio-cultural influences [7]. Goodman (1990, 2008) provided evidence in support of the hypothesis that addictive disorders, including psychoactive substance abuse and BN, have a common, underlying biopsychological process, which includes neurobiological as well as personality factors. According to this hypothesis, the addictive process involves difficulties in three functional systems, namely motivation-reward, affect regulation and behavioural inhibition. Impairment results in various behavioural manifestations such as BN or SUDs [44,45]. Speranza et al. (2012) investigated the extent to which ED patients (without co-morbid SUDs) met Goodman’s (2008) diagnostic criteria for an addictive disorder. They reported that participants with BN met the same proportion of diagnostic criteria as a group with a SUD only (65% vs. 60% respectively). This proportion was significantly higher than those met by the AN-restricting (R-AN) group but not the AN-purging (P-AN) group. No significant correlation was found between impulsivity and addictive personality traits with an addictive ED [46]. Cassin and von Ranson (2007) similarly investigated the association between BED and addictive disorder. They reported that 92.4% of their sample met the DSM-IV criteria for substance abuse/dependence, and 40.5% met Goodman’s (1990, 2008) criteria, which they concluded to be insufficient evidence to reclassify BED as an addiction [47].

##### Personality factors

Evidence from the genetic/familial and addiction models suggests that underlying personality vulnerability factors should be further considered as a potential common causal mechanism for co-morbid EDs and SUDs. Personality vulnerability factors appear to differ depending on the ED subtype [7]. Krug et al. (2009) found an association between family history of alcohol abuse/dependence and highest prevalence of substance use in ED patients with bulimic features and high novelty seeking behaviours. They propose that novelty seeking is a genetically inherited personality trait which predisposes patients to these co-morbid disorders [10]. Similarly, Lilenfeld et al. (1997) reported increased rates of alcohol dependence, drug abuse and dependence (as well as panic disorder, social phobia and Cluster B personality disorders) in first-degree relatives of women with co-morbid BN and substance dependence compared with the first-degree relatives of women with BN without substance dependence. They concluded that anxiety, impulsivity and affective instability may indicate a predisposition for co-morbid BN and substance dependence [48]. In sum, twin and family studies generally do not support a shared genetic link for co-morbid EDs and SUDs, however some evidence suggests that this co-morbidity may be mediated by personality traits such as novelty seeking, which itself may be an inherited trait.

Broad personality sub-types have previously been reported among adolescents and adults with EDs and include (i) high functioning, (ii) emotionally dysregulated, (iii) avoidant/insecure, (iv) constricted/obsessional and (v) behaviourally dysregulated [49]. Findings have been variable regarding the aetiological role of emotional and behavioural dysregulation. Thomson-Brenner et al. (2008) found that the onset of co-morbid substance abuse was most highly associated with the behaviourally dysregulated personality subtype (usually associated with BN), but that ongoing substance abuse was better predicted by previous history of SUD [50]. In contrast however, Benjamin and Wulfert (2005) demonstrated that higher levels of emotional dysregulation were found in those with co-morbid binge eating and alcohol abuse, and impulsivity and anti-social traits were associated rather with either binge eating *or* alcohol abuse [51]. Slane et al. (2013 –in press) found evidence for increased rates of bulimic symptoms and alcohol use difficulties over time in patients with a personality profile characterized by both emotional lability and behavioural dysregulation [52]. Krug et al. (2009) reported an association between novelty seeking and substance use in both those with AN binging/purging type and in those who moved from an AN to a BN diagnosis. The researchers were not able to demonstrate differences between these sub-groups [10]. Current evidence overall, although somewhat inconsistent, more commonly points to an association between underlying personality vulnerability factors and EDs with bulimic features compared with EDs without these features.

Davis and Claridge (1998) researched the extent to which addictive personality traits explained the co-morbidity between EDs and SUDs. They found similar levels of addictiveness across ED subgroups (AN =34 , BN =49) in their sample compared with those previously reported for patients with SUDs. Both ED subgroups were found to have high levels of neuroticism (emotional reactivity, negative affect), which was strongly associated with addictiveness. However for the BN group, addictiveness was also associated with impulsivity and anti-social traits, whereas for those with AN it was associated with introversion [53]. Wilson (2010) argued that there was insufficient support from epidemiological, laboratory, genetic and familial and treatment outcome studies to clinically validate the addictions model [54]. Thus, although there is evidence that some ED patients (particularly those exhibiting binging/purging behaviour) experience their ED as an addiction, evidence for shared addictive personality characteristics is inconsistent and overall there appears to be insufficient empirical evidence in support of the addictions model [7,54,55].

##### Other psychopathology

A related potential mediating factor in the relationship between EDs and SUDs is Attention Deficit Hyperactivity Disorder (ADHD). ADHD is characterized by impulsivity, hyperactivity and inattention, symptoms related to the personality factors previously described, and often present in patients with EDs. Studies have reported co-morbidity between ADHD and EDs and SUDs respectively [56-58]. Kollins (2008), in a review of the literature, reported that co-morbid EDs and ADHD increased the risk for a SUD [57]. Strongest associations have been found between ADHD and EDs with binging behaviours (i.e. BN, BED and EDNOS) [56,58]. The hypothesised pathological mechanisms explaining this co-morbidity include underlying impulsivity and shared genetic risk factors such as neurobiological dysfunction in reward systems [56,58]. With regards to ADHD and substance abuse, Sobanski et al. (2010) identified emotional lability as a predictor of ADHD severity (particularly hyperactivity-impulsivity symptomotology) as well as SUDs in children [59]. ADHD is therefore an important potential mediator and/or aetiological factor in the relationship between EDs and SUDs.

A lifetime history of depression has also been identified as a common underlying factor for EDs and SUDs [60]. Dansky et al. (2000) identified PTSD and MDD as mediating factors in the relationship between BN and alcohol use disorders [19]. Similarly, Measelle et al. (2006) in their sample of adolescent females found that (i) depressive symptoms predicted higher future levels of both eating pathology and substance abuse and (ii) that eating pathology predicted increased future substance abuse but not the inverse. They hypothesised that both pathologies may serve to regulate weight-loss and mood, and distract from negative affect [61].

##### Environmental factors

Environmental factors may impact on the predisposition towards this co-morbid psychopathology. Cumulative childhood trauma can lead to multiple forms of dysregulation, often culminating in psychopathology (including eating disorders and substance abuse disorders) in adolescence or adulthood [62,63]. Baker et al. (2007) identified an association between childhood sexual abuse and co-morbid BN and a SUD [60]. Corstorphine et al. (2007) also found an association between childhood trauma (particularly childhood sexual abuse) and co-morbid substance abuse in a group of ED patients (across ED subtypes, but with a non-significant trend of lower rates in the restrictive AN subgroup). They hypothesised that childhood trauma leads to increased impulsive behaviour, including multi-impulsivity (i.e. BN with concurrent impulsive behaviours such as substance abuse) [64]. However, this relationship appears to be varied and complex, as Rorty et al. (1994) reported that in their sample of BN patients, SUDs were marginally associated with childhood psychological and physical (but not sexual/multiple) abuse only in the presence of a co-morbid personality disorder [65]. The presence of childhood trauma is therefore an important aetiological factor to consider, especially within ED subgroups with bulimic features.

Parental factors associated with co-morbidity include lower parental educational levels, closer maternal relationships, parental modeling of substance abuse or eating disordered behaviours, and maternal emphasis on weight and appearance [8,9,66].

##### Summary

The overall conclusion is that current research indicates *separate* aetiologies and courses for each disorder [7,23,67]. Given the lack of causal evidence, it may be more appropriate to talk about risk factors for the development of co-occurring ED and SUD and to focus research and clinical efforts on the early identification of individuals at risk for this comorbidity.

In considering risk factors for co-morbid EDs and SUDs, developmental processes of adolescence need to be mentioned. Adolescence is associated with increased susceptibility to socio-cultural pressures towards thinness and for and risk-taking behaviour including substance use [8,23]. It is a period involving, among others, increases in appetite, sensation seeking behaviour, emotional reactivity, saliency of social status, parent–child conflict, depression and anxiety [68]. Thus increased social and emotional challenges occur within the context of reduced parental support and immature coping mechanisms. All of this occurring while rapid neurobiological changes are taking place, leaving adolescents vulnerable to unhealthy experimentation or experiences developing into psychopathology [68]. For example, Stock et al. (2002) reported that female adolescents with an ED with purging symptoms used substances in order to relax/improve mood, manage anger, avoid problems and control eating [69]. The high rates of concurrent EDs and SUDs found in adolescents reflect these developmental challenges and highlight the importance of accurate assessment and intervention for this population [9,24,61,70].

### Assessment and diagnosis

Substance use and substance abuse disorders are complicating factors in the assessment, diagnosis and management of EDs. Research has shown that ED patients who abuse substances demonstrate worse ED symptomatology and poorer outcomes than those with EDs alone, and that the presence of an ED in SUD patients leads to greater severity of substance abuse and poorer functional outcomes [4,50]. Sequelae of co-morbidity include severe medical complications [5], longer recovery time from the ED (and/or the SUD) [6,20,71], poorer functional outcomes [4,6], more frequent and/or severe psychiatric co-morbidity [4,6,71], higher rates of suicide/suicide attempts [1,6] and higher mortality rates [72,73]. Further, recent findings suggest that clinicians should be vigilant about the possibility of suicidality in individuals with BN and SUDs because their suicidality risk may be higher than that explained by the SUD alone. Assessing for the presence of suicidality and the level of risk and the temporal order of suicidality, ED and SUD is, therefore, critical [1].

The strongest message conveyed in the current literature is the importance of screening and assessment for co-morbid SUDs and EDs in patients presenting with either disorder [12,74,75]. Once a co-morbid disorder has been identified, a full medical and psychiatric evaluation is recommended, and in the case of AN, patients may need to be medically stabilized before therapeutic treatment can commence for both disorders [8]. One of the challenges to diagnosis is that both ED and SUD patients are often treatment resistant and may experience shame and/or guilt, leading to reluctance to report ED or SUD symptoms [8,12,75]. Drug and alcohol use can also have an influence on features that are more specific to the assessment of EDs, such as weight, appetite and dietary restriction, thus complicating the diagnostic process [75]. Collateral information is therefore key when assessing patients with EDs, while the importance of a direct but non-judgmental approach during assessment is also emphasized [12,74].

The use of standardized screening or assessment questionnaires is advisable [75,76]. Such screening tools could be used for assessing risk for both disorders in primary care [74]. Black and Wilson (1996) found the Eating Disorder Examination – Questionnaire (EDE-Q) to be a valid screening tool to identify ED symptoms amongst a clinical sample of substance abuse patients, particularly for diagnosing BN and identifying low level ED behaviours where further assessment is indicated [77]. For a review of screening tools for the risk/presence of EDs see Grilo et al. (2002) [76]. According to Conason et al. (2006) brief screeners for the presence of alcohol abuse/dependence designed for use in primary care settings include the CAGE questionnaire (cut down, annoyed, guilty, eye opener) and the TWEAK test (tolerance, worried, eye-opener, amnesia, cut-down) [74,78,79]. Longer instruments include the Michigan Alcoholism Screening Test (MAST) and the Alcohol Dependence Scale (ADS) [80,81]. Conason et al. (2006) also recommend the following screening tools for the presence of other substance disorders: the Drug Abuse Screening Test (DAST); the Two Item Alcohol and Drug Screening Questions; and the Drug Screening Questionnaire (DSQ) [74,82-84].

Conason et al. (2006) have argued that interviewing is the most effective assessment technique for diagnosing co-morbid EDs and SUDs [74]. They recommend taking a detailed substance abuse history, including current and lifetime substance use and periods of greatest severity. Such a history should also incorporate detailed information on the function and patterns of substance use. Questions should specifically explore the misuse of substances as weight loss mechanisms, for example, caffeine, tobacco, insulin, thyroid medications, stimulants or over the counter medications (laxatives, diuretics) used for metabolism restriction, caloric restriction, appetite suppression or purging [11,36,37]. Similarly, the role of alcohol or psychoactive substances in emotional regulation should be explored, for example, the use of alcohol, opiates or cannabis for the relief of anxiety, depression, guilt or shame, or for emotional reward [27]. Wolfe and Maisto (2000) highlight the importance of a behavioural assessment (including questionnaires, self-monitoring, role play and collection of collateral information) in order to explore the functional relationship between substance use patterns and ED behaviours. Precipitants, concomitant affective states and consequences of each set of behaviours should be considered as this will aid teasing out specific risk factors as well as an understanding of the mutual influence of behaviours and emotional states [23].

While the current psychiatric nosology for EDs utilises a symptom based approach, recent research has also focused on an alternative classification system for EDs based on co-morbid psychopathology and associated features [3]. The proposed classification systems include (i) dietary versus dietary-negative affect EDs (where the ED is characterized by either dietary restraint, negative affect or a combination of both), (ii) under-controlled versus over-controlled EDs (which considers patterns of mood disturbances, anxiety and impulsivity) and (iii) low psychopathology EDs [3]. Such a diagnostic approach may provide a structured way of assessing risk factors, patterns and maintaining factors of substance use in ED patients.

The following section considers the management of co-morbid EDs and SUDs including an outline of different treatment modalities.

### Management

Some of the pertinent questions in the treatment of co-morbid EDs and SUDs include how to ascertain the presence of a co-morbid disorder, whether to treat the disorders concurrently, and if not, which disorder to address first [12,76]. One difficulty is that treatment studies for EDs and SUDs often exclude patients with dual diagnoses making research evidence on effective management strategies for this population extremely scarce [12,85]. Nevertheless, there are a number of important considerations. Firstly, sequential treatment may lead to an increase/relapse of symptoms of one of the disorders as symptoms of the other disorder improve [4,8,86]. Secondly, symptoms of the disorder not being treated may interfere with recovery from the disorder for which treatment is underway [87,88]. Thirdly, inadequate management of both disorders can also increase relapse rates in symptoms of one or both [8,86]. An additional consideration is the presence of other co-morbid psychiatric diagnoses such as anxiety and depression which may need to be simultaneously managed in these patients [4].

Despite the paucity of treatment outcome studies, some researchers suggest that treatments which target aetiological factors common to both disorders are effective, for example addressing difficulties with emotional regulation in concurrent binge eating and substance use disorders [89]. Woodside and Staab (2006) recommend that when there is a current SUD, patients should undergo detoxification prior to ED treatment, and where possible this should be combined with ED treatment, for example in a residential treatment facility [90]. Overall, the literature indicates that the ED and SUD should be addressed simultaneously [4,76,86]. CASA (2003) recommend programmes which include treatments focused on substance abuse and EDs specifically, as well as individually tailored combinations of personal, group and family therapy provided by a multi-disciplinary team [8]. General treatment principles for eating disorders such as establishing a trusting, collaborative therapeutic relationship and avoiding power struggles should be followed [91]. Several treatment modalities are considered below.

#### Medical stabilisation

Patients with AN with particular medical indications such as critically low BMI, blood chemistry imbalances, dehydration, irregular cardiac function and heart rate and blood pressure abnormalities, may require hospitalisation and/or nutritional rehabilitation [92,93]. The rate and severity of change in physiological functioning are important to consider, for example less severe but rapid changes may necessitate hospitalization [93]. For patients with AN, weight restoration may be important prior to the commencement of psychological treatment because the effects of starvation on affect and cognition can interfere with therapy [93].

ED patients who abuse laxatives require immediate laxative withdrawal because of the physical danger of laxative abuse, and the increased risks of mortality [94]. Management recommendations include immediate cessation of laxative use and medication to promote bowel function if necessary, encouragement of high fibre intake and exercise, and psycho-education. Psycho-education should cover aspects relating to the physiological effects of laxative abuse, the effects of laxative withdrawal, the physiology of normal bowel functioning and cognitive distortions surrounding laxative use, including that it does not result in weight loss [12,94]. Alternatively, (and especially for patients with a flaccid bowel), stimulant laxatives may be replaced by osmotic laxatives such as lactulose to promote bowel function [12,94]. Once all laxatives have been discontinued, a programme of desensitisation to laxative use may be implemented [94]. Colton et al. (1999) have reported a success rate of 57% short to medium term laxative abstinence in their sample following an inpatient laxative withdrawal programme [94].

#### Pharmacotherapy

Research indicates that pharmacotherapy alone should not be the primary treatment for AN [76,95], however anti-depressants (particularly SSRIs) have been effectively used to treat both BN patients as well as patients with an alcohol use disorder with co-morbid major depression [38,76,95]. Some evidence has also been found to support the use of opioid antagonists in the treatment of both EDs and AUDs [38]. Pharmacotherapy may be indicated in combination with therapeutic interventions [8].

#### Psychological treatments

Various psychological interventions have been used in the treatment of co-morbid EDs and SUDs, ranging from individual to group and family therapy. Common features across interventions include psycho-education regarding the aetiological commonalities, risks and sequelae of concurrent ED behaviours and substance abuse, dietary education and planning, cognitive challenging of eating disordered attitudes and beliefs, building of skills and coping mechanisms, addressing obstacles to improvement and the prevention of relapse [76,89,96].

There is evidence for the efficacy of various forms of CBT, including self-help CBT programmes, in the treatment of EDs (particularly BN), however the efficacy of CBT in the presence of co-morbid SUD has not been examined [8,74,76,86,95,97-99]. Sinha and O’Mally (2000) and Grilo et al. (2002) nevertheless suggest that for the treatment of EDs and alcohol abuse, a CBT approach which targets both pathogenic eating behaviours as well as alcohol use is likely to be effective [38,76]. They identify particularly useful strategies such as self-monitoring, identification of high risk situations and coping skills to manage emotions or situations which may trigger loss of control. Often a “stepped-care” approach is recommended where patients begin with self-help CBT and if necessary proceed to guided self-help interventions or to group or individual therapy [100].

Motivational interviewing (MI) can be used prior to CBT intervention and aims to increase the likelihood of a patient engaging with and continuing therapy by improving insight into the problem, building commitment and increasing intrinsic motivation for change [100]. MI combined with therapist-client feedback regarding the progress of symptom improvement (compared to the norm) and difficulties with achieving target behaviours is called motivational enhancement therapy (MET), and can be used as an individual or adjunctive treatment [100]. Dunn et al. (2006) report mixed evidence for MET in the treatment of EDs. They found that one session of MET used as an adjunct to a CBT self-help programme in a group of patients with BN or BED led to increased readiness to change bingeing behaviours, but did not lead to change in eating attitudes, frequency of bingeing and compensatory behaviours or treatment compliance [100]. No studies have examined the efficacy of MET with co-occurring EDs and SUDs.

Dialectical behaviour therapy (DBT) has been investigated as a treatment for co-morbid EDs and SUDs [76]. Grilo et al. (2002) suggest that DBT, even when not focusing on ED behaviour specifically, may reduce ED symptoms in patients with BN and BED, because it teaches emotional regulation strategies and coping behaviours, and these disorders have been associated with emotional and behavioural dysregulation [76]. Courbasson et al. (2012), in a study comparing the efficacy of DBT with treatment as usual (TAU) (which consisted of a combination of motivational interviewing, CBT and relapse prevention strategies) for patients with co-morbid EDs and SUDs, reported improved retention rates for the DBT group compared with the TAU group (87% vs. 20% post-intervention and 60% vs. 20% at 3- and 6- month follow-ups) [85]. Their results also provide preliminary positive evidence for cognitive and behavioural treatment outcomes in the DBT group, including improved ED behaviours and attitudes, reduced rate and severity of substance use, greater regulatory capacity for negative emotions and improvement in depressive symptoms. Nearly all improvements were present post-intervention and sustained at 3- and 6- month follow-up [85]. Courbasson et al. (2011) reported similar results for the use of mindfulness-action based cognitive behavioural therapy (MACBT) (which includes teaching mindfulness and mindful eating, increasing emotional regulation skills, providing psycho-education, encouraging balanced physical activity and focusing on strengths) for a group of patients with BED and co-morbid SUDs. They describe fewer objective binge eating episodes, reductions in severity of drug addiction and improvements in disordered eating attitudes and depressive symptoms post treatment [89].

Some researchers also report efficacy of 12 step programmes for the treatment of SUDs, especially alcohol abuse/dependence [12,87]. Such programmes could run concurrently and work effectively with ED treatment [87]. Long term individual psychotherapy has also been recommended in the treatment of co-morbid EDs and SUDs, however, this is thought to be more appropriate once recovery from SUD has been maintained for a period of time. CBT type therapies are recommended during or after substance use recovery treatment [87].

Additional treatments that are recommended for EDs include cognitive analytic therapy, interpersonal psychotherapy, focal psychodynamic therapy and family therapy (especially for adolescents) [8,92,93,95].

#### Inpatient vs. outpatient treatment

The 2004 NICE clinical guidelines indicate that where possible ED patients should be treated on an outpatient basis for at least 6 months, except where severe co-morbid substance abuse is likely to interfere with outpatient treatment efficacy [95]. Indicators for hospitalisation, partial hospitalisation, residential or intensive outpatient treatment may depend on general medical and psychiatric complications (such as depression or suicidality), symptom severity and previous course of the illness [92].

Franko et al. (2005) found that both inpatient and outpatient treatments were effective at addressing AUD in patients with an ED [20]. Residential treatment programmes have been found to be effective in the treatment of AN and BN both post-treatment and at 3–4 year follow-up [101,102]. Treatment for EDs may also take place within substance abuse treatment programmes, however not all such programmes are equipped with adequate knowledge or resources [93]. Killeen et al. (2011) found that of the addiction treatment centres in their sample that screened for the presence of EDs, 67% of centres reported that they admit low level ED patients, and 21% reported that they treat EDs [75]. Where inpatient or residential treatment is preferred, follow-up treatment in the form of individual or group therapy, support groups or 12 step programmes is vital to preventing relapse of both disorders [87,93].

## Conclusion

SUDs are prevalent in patients with EDs and are more commonly seen in ED patients with bulimic features. Substances of abuse range from alcohol to illicit drugs as well as over the counter or prescribed medications. Alcohol and psychoactive drugs may be used for emotional regulation or as part of a pattern of impulsive behaviour. Caffeine, tobacco, laxatives, stimulants, thyroid medications and insulin may be misused as weight-loss aids. While EDs and SUDs have shared and associated features and risk factors, the literature indicates a number of distinct differences in the aetiology and course of both disorders. The importance of screening ED patients who present for treatment for SUDs, and visa versa, is emphasized in the literature. The functional relationship between the ED and substance of abuse varies across ED subtypes and needs to be carefully assessed. There is a paucity of treatment studies for the management of co-morbid EDs and SUDs, however, the available literature suggests that where possible, both disorders should be treated simultaneously using a multi-disciplinary approach. The need for hospitalization or inpatient treatment depends on general medical and psychiatric considerations. Patients may need to be medically stabilized before therapeutic treatments are employed. Emphasis should be placed on building a collaborative therapeutic relationship. While CBT has been frequently used in the treatment of co-morbid EDs and SUDs, there are no randomized controlled trials of its use in this context. More recently evidence has been found for the utility of DBT in reducing both ED and substance use behaviors, with these improvements sustained over time.

This review provides a broad overview of the prevalence, aetiology, assessment and management of co-morbid EDs and SUDs. It integrates and discusses current research and attempts to delineate the functional relationship between ED subtypes and various substance use/dependence/abuse. It is by no means exhaustive and future research would benefit from a meta-analysis of all the current research in order to better understand the relationships between these two commonly co-occurring disorders.

## Competing interests

All authors declare that they have no competing interests.

## Authors’ contributions

CG, SS and GPJ were all involved in the conception and design of the study. CG wrote the first draft of the manuscript. SS and GPJ were all involved in the critical revision of the manuscript. CG, SS and GPJ approved the final manuscript.

## Pre-publication history

The pre-publication history for this paper can be accessed here:

http://www.biomedcentral.com/1471-244X/13/289/prepub

## Supplementary Material

Additional file 1: Table S1 Summary of prevalence studies: Co-morbid eating disorders and substance use disorders. Click here for file
