# Supplementary material for: A clinical approach to the assessment and management of co-morbid eating disorders and substance use disorders
Source: BMC Psychiatry. 2013 Nov 7;13:289. doi: 10.1186/1471-244X-13-289 (PMC4226257; doi:10.1186/1471-244X-13-289)
Supplement: Additional file 1: Table S1 — Summary of prevalence studies: Co-morbid eating disorders and substance use disorders. [file 1471-244X-13-289-S1.doc]

| **Additional file 1: Table S1.** Summary of prevalence studies: Co-morbid eating disorders and substance use disorders | | | | | | | | | | |
| --- | --- | --- | --- | --- | --- | --- | --- | --- | --- | --- |
| **Authors, date**  **Journal** | **Sample Characteristics** | **Procedure , Aim** | **Tools** | | | **Main Results** | **Prevalence** | | **Possible Confounding Factors** | |
| [6] Glasner-Edwards et al. (2011)  Eating Disorders | 526 Metham-phetamine (MA) dependent adults  316 Female  210 Male | Longitudinal study, Assessment interviews at baseline, treatment discharge, 3 year follow-up.  Statistical analysis of ASI scores across all three time periods.  Aim: To examine psychiatric, substance use, and functional outcomes of MA users with BN 3 years after treatment for MA dependence | Addiction Severity  Index (ASI), Life Experience Timeline Interview (LET), Mini-International Neuropsychiatric Interview (MINI) | | | BN was observed in 2.4% of the sample 3 years after treatment for MA dependence and was associated with poorer MA use outcomes, an increase in the use of health services and greater functional impairment | 2.4% of the sample | | Co-morbid psychiatric diagnoses  Socio-economic difficulties | |
| [7] Harrop and Marlatt (2010)  Addictive Behaviours | 13 peer-reviewed articles | Systematic review  Searches on Psych Info and Medline |  | | | High levels of co-morbid EDs and SUDs in both clinical and community samples  The presence of one disorder increases the risk for the other disorder by over 4 times  ED symptomotology seems more indicative of SUD than ED subtype | 17-46% | | Differing classification/diagnostic criteria for ED and SUD across studies | |
| [9] Denoth et al. (2011)  PloS One | 33,185 15–19 year old adolescents  16,911 female  16,904 male | Cross-sectional study  Self-report questionnaires  Statistical analysis  Aim: To examine the distribution of gender-stratified BMI, eating attitudes and substance use amongst teenagers with weight abnormalities, ED and SUD; to examine the influence of family, peer-related and psychosocial factors on compulsive behaviour | European School Survey Project on Alcohol and Other Drugs questionnaire, Eating Attitude Test 26 (EAT-26) | | | Recent drugs use was more frequent in overweight and underweight adolescents, especially in females | 20-40% greater use/ abuse of substances associated with abnormal weight control | | Differences in parental control and  economic dependence across geographical areas | |
| [10] Krug et al. (2009)  Psychiatry and Clinical Neuro-sciences | 371 female ED patients | Cross-sectional study, Structured, clinical interviews  Statistical analysis  Aim: To assess lifetime substance abuse, family history of alcohol dependence/abuse and novelty seeking in 3 ED groups (AN-restrictive; AN-binge eating/purging; AN to BN cross-over) | Interview modelled after the Structured Clinical Interview for DSM-IV (SCID-I), Novelty Seeking subscale of the Temperament  and Character Inventory–Revised (TCI-R) | | | Apparent greater prevalence of lifetime substance abuse among patients with AN with bulimic features  Higher novelty seeking scores may be associated with diagnostic cross-over from AN to BN | Lifetime substance abuse:  AN-R: 8.8%  AN-BP: 26.3%  AN-BN: 35.2% | | Co-morbid psychiatric diagnoses  Family history of substances other than alcohol | |
|  |  |  |  | | |  |  | |  | |
| **Additional file 1: Table S1.** Continued | | | | | | | | | | |
| **Authors, date**  **Journal** | **Sample Characteristics** | **Procedure , Aim** | **Tools** | | | **Main Results** | **Prevalence** | | **Possible Confounding Factors** | |
| [11] Krug et al. (2008)  Drug and Alcohol Dependence | 879 ED patients  785 Healthy controls | Case-control design  Structured diagnostic face-to-face or phone interview  Self-report questionnaires  Statistical analysis  Aim: To assess the differences in co-morbid lifetime and current SU between ED  patients and healthy controls | Substance Use Subscale of the Cross Cultural (Environmental) Questionnaire, EATATE phenotype interview,  General health questionnaire-28,  Eating Attitudes Test (EAT) | | | Higher prevalence of smoking, and drug use (but not alcohol use) in the ED patients compared with the control group  BN and AN-BP groups had more frequent tobacco and drug use, and the EDNOS group reported more frequent alcohol use compared with other ED subgroups | ED patients:  Tobacco 47.5%  Alcohol 34.1%  Drugs 61.3%  Controls:  Tobacco 35.1%  Alcohol 26.9%  Drugs 43.3% | | Co-morbid psychiatric diagnoses | |
| [13] Root et al. (2010a)  International Journal of Eating Disorders | 731 female AN patients  328 AN-restricting, 184 AN purging, 109 AN binging, 110 lifetime AN and BN | Cross-sectional study  Semi-structured clinical interview  Self-report questionnaires  Blood sample data  Statistical analysis  Aim: To examine the prevalence of SUD in subgroups of ED patients | Structured Inventory of Anorexia Nervosa and Bulimic Syndromes, SCID-I | | | Differing prevalence of SUD across AN subtypes  Higher prevalence amongst the ANBN group  Purging associated with SU  Prevalence of SUD differed across lifetime binge eating status | Total sample  Alcohol use/ dep 19.8%  Lifetime drug use 25.9%  Lifetime drug abuse/ dep 13.8% | | Co-morbid psychiatric diagnoses (i.e. depression, anxiety)  Negative affect | |
| [14] Root et al. (2010b)  Psychological Medicine | 13 297 female participants from the Swedish Twin Registry  No ED=94%, AN= 3%  BN = 2%  ANBN =1%  BED = <1% | Case-control design  Web based questionnaires or computer-assisted telephone interviews  Statistical analysis  Aim: To examine the association between ED and SUD in a large population based sample | SCID-I | | | Presence of an ED was associated with greater SU compared with the control group (no ED)  BN and ANBN groups had > alcohol use/ dep than AN group; Increased illicit drug use in the AN group  ANBN group had greatest diet pill use followed by the BN group | Across ED subgroups:  Alcohol use/ abuse 12-24%  Regular drug use 0-11%  No ED:  Alcohol use/ abuse 6%, Regular drug use <1-2% | | Co-morbid psychiatric diagnoses (i.e. depression, anxiety)  Negative affect | |
| [15] Calero-Elvira et al. (2009)  European Eating Disorders Review | 16 peer reviewed articles, comprising data on 42 236 individuals | Meta-analysis, Searches on Medline, PsycINFO, Web of Science and CINAHL, Statistical analysis  Aim: To examine prevalence of DU in ED sample compared to HC, to perform a meta-analysis on the current literature |  | | | Highest risk for SU in BN group followed by BED group  Both significantly higher SU than HC group  No significant difference between AN group and HC group | Not assessed | | Variability of classification/diagnosis of SU and ED across studies | |
| [16] Baker et al. (2010)  International Journal of Eating Disorders | 1206 monozygotic and 877 dizygotic  female twins | Cross-sectional study Structured clinical interviews  Statistical analysis. Aim: To examine the prevalence, chronology, and possible shared familial risk between SUD and ED symptomotology | Adapted version of the Structured Clinical Interview for DSM-III-R (SCID) | | | No significant difference in SUD between AN and BN groups Compared with controls, AN and BN groups at increased risk of alcohol use disorder and to smoke regularly  BN more likely to have an illicit drug use disorder | Co-morbid AN and SUD 17-52%  Co-morbid BN and SUD 18-45% | | Some variability in diagnostic criteria for ED across the sample | |
|  |  |  |  | | |  |  | |  | |
| **Additional file 1: Table S1.** Continued | | | | | | | | | | |
| **Authors, date**  **Journal** | **Sample Characteristics** | **Procedure , Aim** | **Tools** | | | **Main Results** | **Prevalence** | | **Possible Confounding Factors** | |
| [17] Holderness et al. (1994)  International Journal of Eating Disorders | 51 research studies | Literature review  Aim: To examine the associations between ED and SU/SUD; to examine the mechanisms explaining the ED- SU/abuse |  | | | BN and patients with bulimic behaviours have greater SU than those with AN  AN group with bulimic features report more SU/abuse than  AN-R groups | Alcohol/ drug abuse/ dep  BN 0-55%  AN 3-13%  ANBN 6.7-43% | | Some variability in the criteria for SUD across studies | |
| [18] Piran and Robinson (2006)  Addictive behaviours | 526 female university students | Cross-sectional study  Self-report questionnaire  Statistical analysis  Aim: To examine the association between ED behaviours and SU/ abuse | The Women’s Health Survey | | | Associations found between (1) severe alcohol use and BE; (2) stimulant drugs and dieting with purging; (3) tobacco use, prescription medication abuse and BE | Substance use:  No ED 2-41%  Binge only 0-42%  Diet only 3-41% | | No measure of BE severity  Stringent definition of severe alcohol use | |
| [19] Dansky et al. (2000)  International Journal of Eating Disorders | 3006 females participants | Cross-sectional study  Probability sampling  Telephonic interviews  Statistical analysis  Aim: To examine the nature of the relationship between BN and alcohol use/ dependence | National Women’s Study (NWS) Telephonic interview | | | Alcohol abuse greater in women with BN than those without BN or BED (HC), only when MDD and PTSD controlled for | Alcohol abuse:  BN 31%, HC 18.8%  Alcohol dep: BN 13.2% HC 4.3% | | Non-standardised diagnostic measures | |
| [20] Franko et al. (2005)  International Journal of Eating Disorders | 136 female AN patients  110 female BN patients | Longitudinal study  Telephone screening  Semi-structured clinical interviews  Statistical analysis  Aim: To examine the mutual influence of alcohol use disorders (AUD) and ED | Eating Disorders Longitudinal Interval Follow-Up Evaluation (LIFE-EAT II), Research  Diagnostic Criteria (RDC) for Alcohol use disorder, DSM-IV Global Assessment of Functioning Scale (GAF),  The Structured Interview for DSM-III Personality Disorders (SIDP) | | | No significant difference between AN and BN groups for lifetime history of AUD  The influence of ED on AUD is greater than the reverse  Poor psycho-social functioning and history of SU predicted onset of AUD for AN and BN groups  Depression, elevated body image concern and vomiting predicted AUD in the AN group | Lifetime history of AUD 27% | | Variability in diagnostic criteria for ED and AUD | |
| [21] Piran and Robinson (2011)  Journal of Health Psychology | 1043 female participants, 526 university students, 517 from community settings | Cross-sectional study Self-report questionnaire  Statistical analysis  Aim: To examine patterns of association between eating disordered behaviour and SU | The Women’s Health Survey | | | In both samples, significant association between a cluster of binging, dieting and purging with binge drinking and cocaine use, and between dieting and purging with amphetamine/stimulant use | Not assessed | | Co-morbid psychiatric diagnoses | |
| [22] Stewart et al. (2000)  Psychology of Addictive Behaviours | 176 female university students | Cross-sectional study  Self-report questionnaires  Statistical analysis  Aim: To examine the relationship between dietary restraint and alcohol use (AU) | Demographics and drinking behaviour measure, Restraint Scale (RS) | | | Dietary restraint was positively associated with 4/5 drinking behaviour measures | Not assessed | | Not stated | |
|  |  |  |  | | |  |  | |  | |
| **Additional file 1: Table S1.** Continued | | | | | | | | | | |
| **Authors, date**  **Journal** | **Sample Characteristics** | **Procedure , Aim** | | **Tools** | **Main Results** | | **Prevalence** | | **Possible Confounding Factors** | |
| [24] Arias et al. (2009)  Substance Abuse: Research and Treatment | 177 adolescents in treatment for SUD  59 female  118 male | Cross-sectional study  Telephonic screening interview  Diagnostic interview  Statistical analysis  Aim: To examine the relationship between ED symptoms and severity of AU | | Teen Addiction Severity Index(T-ASI), Alcohol Consumption Questionnaire (ACQ), Diagnostic  Interview Schedule for Children (DISC-IV) | Number of ED symptoms correlated with increased alcohol consumption | | 26.4% of the sample had 1 or more ED symptoms  0.6% of the sample had a diagnosis of BN | | Co-morbid psychiatric diagnoses (including SUD other than alcohol, nicotine or marijuana) | |
| [25] Gadalla and Piran (2007)  The Journal of Treatment & Prevention | 36 984 participants from the Canadian Community Health Survey (CCHS) on Mental-Health and Well-being  20 2011 female, 16 773 male | Cross-sectional study  Secondary analysis of data from the CCHS - multistage stratified cluster sample  Computer assisted personal interviewing method  Statistical analysis  Aim: To examine the co-morbidity between ED and SU | | EAT-26,modules from the Composite International Diagnostic Interview (CIDI-SF), questions based on Canada’s Alcohol and Other Drugs Survey (CADS) | Whole sample: ED risk associated with alcohol interference and amphetamine use  Women sample: ED risk associated with illicit drug use, dependence and interference and number of substances used | | ED risk and 12 month alcohol dep/interference: women 3-3.8%, men 8.3-9.1%; ED risk and 12 month illicit drug dep/interference: women 21-24%, men 0-1.2%  (excl cannabis) | | Lack of diagnostic information on ED behaviour  Personality factors  Experience of trauma | |
| [26] Piran and Gadalla (2006)  The Journal of Treatment & Prevention | 20 2011 female participants from the Canadian Community Health Survey (CCHS) on Mental-Health and Well-being  15.9% 15–24 yrs, 37.3% 25–44 years, 46.8% 45 years+ | Cross-sectional study  Secondary analysis of data from the CCHS - multistage stratified cluster sample Computer Assisted Personal interviewing method  Statistical analysis  Aim: To examine the co-morbidity between ED and SU | | EAT-26,modules from CIDI-SF, questions based on the CADS | Whole sample: ED risk was associated with alcohol dependence and interference and lifetime illicit drug use and dependence | | ED risk and 12 month alcohol dep/ interference 1.1-9.3%; ED and lifetime illicit drug use 21.4-57.7%; ED risk and 12 month illicit drug dep/ interference  0-9.8% (excl cannabis) | | Lack of diagnostic information on ED behaviour  Personality factors  Experience of trauma | |
| [27] Stewart et al. (2006)  Journal of Health Psychology | 58 women receiving treatment for an alcohol problem | Cross-sectional study, Self-report questionnaires, Qualitative interviews, Statistical analysis  Aim: To examine the prevalence and characteristics of binge eating behaviours; to examine common underlying motivations for binge eating and drinking | | 10-item Brief Michigan Alcoholism Screening Test (B-MAST), 12-item Binge Scale, 42-item Inventory  of Drinking Situations, Inventory of Binge Eating Situations | 71% of the sample self-identified as binge-eaters (BE)  BE were younger, drank more frequently, and more often for emotional relief compared with non-BE, BE and heavy drinking appeared to serve similar functions in individuals | | 71% self-identified as binge-eaters, 90% of which were classified as ‘severe’ binge eaters | | Self-diagnosis as ‘binge-eater’  Co-morbid psychiatric diagnoses such as PTSD | |
| [28] Jeffers et al. (2013)  Appetite | 705 psychology students  61.3% female  38.7% male | Cross-sectional study, Survey, Non-random sampling, Statistical analysis. Aim: To assess the prevalence of prescription stimulant use for weight loss; to examine the relationship to other health jeopardising behaviours | | Online questionnaire compiled by researchers | 11.7% of the sample used prescription stimulants to lose weight, Motivation included appearance, emotion and stress regulation This group reported poorer coping ability, lower self-esteem and more additional ED and unhealthy weight-loss behaviours | | 11.7% of the total sample  13.1% females and 9.7% males | | Co-morbid psychopathology (i.e. depression, anxiety, ADHD, ED)  Co-morbid alcohol and elicit substance use  Body image | |
|  | | | | | | | | | | |
| **Additional file 1: Table S1.** Continued | | | | | | | | | | |
| **Authors, date**  **Journal** | **Sample Characteristics** | **Procedure , Aim** | | **Tools** | **Main Results** | | | **Prevalence** | | **Possible Confounding Factors** |
| [29] Prior et al. (1996)  International Journal of Eating Disorders | 331 female ED patients in an outpatient clinical setting  51 AN (binge-eating/ purging type)  280 BN (purging type) | Cross-sectional study Clinical interviews  Self-report questionnaires  Statistical analysis  Aim: To investigate the relationship between history of laxative abuse (LA) to eating and associated attitudes, impulsivity and personality pathology | | Diagnostic survey for Eating Disorders-Revised (DSED-R), Eating Disorder Inventory (EDI), Millon Clinical Multi-axial Inventory (CMI) | 58.3% of both groups had a lifetime history of LA  LA unrelated to ED diagnostic category  LA predictive of perfectionism and avoidant personality features  LA predictive of particular types of psychopathology/ personality characteristics as a function of ED diagnosis | | | 58.3% of total sample  51% of AN group  59.6% of BN group | | Sexual abuse history  Personality pathology |
| [30] Bryant-Waugh et al. (2006)  International Journal of Eating Disorders | 201 ED patients at an outpatient ED treatment service  199 female  2 male  10.4% AN, 32.8% BN, 56.7% EDNOS diagnosis | Cross-sectional study  Self-report questionnaires  Structured clinical interview  Physical measurements  Statistical analysis  Aim: To examine the prevalence of laxative abuse among adult ED patients; to explore LA across ED diagnostic categories; to describe demographic and psychobehavioural profiles of laxative abusers (LAs) and to identify predictors | | Beck Depression Inventory(BDI), several Stirling Eating Disorder Subscale scores (SEDS), several subscale scores from the Eating Disorder Examination (EDE), | 26.4% had abused laxatives in the previous month, prevalence similar across diagnostic categories  LA predicted anorexic behaviours and cognitions, weight/ shape concerns  LA associated with depression and self-directed hostility  LA predicted by depression and anorexic behaviours | | | 26.4% had abused laxatives in the previous 3 months  AN (N=10)  BN (N=34 )  EDNOS (N=62) | | Personality factors/ pathology |
| [32] Leaf et al. (2012)  American Journal of Kidney Disease | 1 ED patient with co-morbid nephrolithiasis associated with laxative abuse (LA) | Case Study  Literature review, Medline search  Aim: To present a case of nephrolithiasis associated with LA, to explain the paucity of such cases, to review the literature, | |  | Most ED patients are likely to be protected from kidney stone formation by the hypocalciuric effect of extracellular fluid volume depletion and increased proximal tubular sodium reabsorption  ED patients with underlying idiopathic hypercalciuria may be at increased risk of kidney stone formation | | | Not assessed | | Underlying hypercalciuria as an additional risk factor |
| [33] Lachenmeyer et al. (1988)  International Journal of Eating Disorders | 1261 adolescents  Lower SES:  328 female  384 male  Higher SES:  306 female  243 male | Cross-sectional study  Self-report questionnaires  Statistical analysis  Aim: To investigate laxative abuse (LA) amongst adolescents of differing SES, to assess the relationship between LA and ED | | EAT, Binge Eating Questionnaire (BEQ) | 3.5% of the sample used laxatives for weight control  No gender differences found  LA twice as high among high SES adolescents  LA highly correlated with use of diuretics  Association between BN and LA | | | 3.5% of the sample abused laxatives for weight control  45% of LAs showed no accompanying ED | |  |
| [34] Mascolo et al. (2011)  International Journal of Eating Disorders | Not stated | Literature review  Aim: To illustrate the  pathophysiologic effects of diuretics and purging on a patient with BN, to clarify the role of diuretics during refeeding | |  | Purging behaviours lead to volume depletion and increased aldosterone production Aggressive rehydration of BN patients in the context of hyperaldo- steronism leads to avid salt retention and the development of marked amounts of edema | | | BN patients: 60.6% abused laxatives, 33.9% abused diuretics | |  |
|  | | | | | | | | | | |
|  | | | | | | | | | | |
| **Additional file 1: Table S1.** Continued | | | | | | | | | | |
| **Authors, date**  **Journal** | **Sample Characteristics** | **Procedure , Aim** | | **Tools** | **Main Results** | | **Prevalence** | | **Possible Confounding Factors** | |
| [35] Edler et al. (2007)  International Journal of Eating Disorders | 111 female Purging Disorder (PD) patients | Cross-sectional study  Self-report questionnaires  Clinical interviews  Statistical analysis  Aim: To examine whether  purging frequency or the use of multiple  purging (MP) methods is associated with ED severity | | Barratt Impulsiveness Scale (BIS-11), BDI, Body Shape Questionnaire (BSQ), EDE, State-Trait Anxiety Inventory  (STAI), SCID-I, Structured Clinical Interview for  DSM-IV Axis II Personality Disorders (SCID-II), Three Factor Eating Questionnaire (TFEQ) | MP methods associated with greater ED severity  Purging frequency associated  with increased binge frequency and general psychopathology | | Not assessed | | Co-morbid laxative abuse | |
| [37] Polonsky et al. (1994)  Diabetes Care | 341 Female insulin-dependent diabetes mellitus (IDDM) out-patients | Cross-sectional study  Self-report questionnaires  Statistical analysis  Aim: To examine the prevalence intentional insulin omission, to examine its relationship  to disordered eating, attitudes toward diabetes, other psychosocial factors, long-term complications,  and glycemic control | | Bulimia Test-Revised (BULIT-R), Brief Symptom Inventory (BSI), Hypoglycemia Fear Survey, Problem Areas in Diabetes  Survey (PAID), Self-Care Inventory (SCI) | Insulin omission is common and may have severe medical consequences  A strong association found between omitting and eating disordered behaviour Approximately 50% of omitters did so for weight-management This subgroup displayed greater psychological distress, more frequent omission, poorer glycemic control and more complications | | 30.5% of the sample reported intentional insulin omission  8.8% reported frequent insulin omission | | Other diabetes specific factors | |
